# Introduction: extraordinary issue II: coronavirus, crisis and communication

**DOI:** 10.1177/1329878X20969949

**Published:** 2021-02

**Authors:** Craig Hight

**Affiliations:** The University of Newcastle, Australia

In this, our second concurrent issue dealing with the ongoing implications of COVID-19 for media and communication, we continue with the presentation of a first slice of commentaries and initial findings from research approaches addressing the implications of the virus.

We present two subsections of this work.

## Australia’s first wave

The initial responses of each country to the unfolding COVID-19 pandemic have been varied, and much has depended on local circumstances and recent history. The first months of the virus’ emergence quickly revealed the level of preparedness of national health systems and vastly different leadership capabilities of national and regional governments. From the initial outbreak reported in China in December 2019, then cases appearing in neighbouring countries and those connected through international air travel, to the declaration of a global pandemic by the World Health Organization in late January 2020, each local population has been confronted with a number of challenges. Citizens have faced rapidly changing health advice playing out in unexpected scenarios in everyday lives, confusion over how rigorously to follow government initiatives and dictates and a wider realisation of the vulnerabilities of modern life to a relentless virus. In Australia’s case its political, cultural and economic responses were initially complicated by a period of disruption and recovery from a disastrous spate of bushfires over the 2019–2020 summer, events widely reported as ‘unprecedented’. Perhaps more crucially, it became apparent that the local population had little cultural memory of dealing with viruses as disruptive as COVID-19. The global influenza pandemic of 1918 (H1N1 virus), a familiar reference point of media commentary, appeared too distant; the 1937 polio epidemic was rarely mentioned; and memories of the HIV/AIDS pandemic had seemingly receded since the homophobic hysteria of the 1980s. The more recent severe acute respiratory syndrome (SARS) outbreak of 2003–2004, which proved to be such an important touchstone for national anti-virus responses in South-East Asia, was always assumed by Australians to be localised ‘overseas’ and did not transpire as a disruptive agent at the national level. Australians, along with many other nations, revealed themselves to be lacking an imagination for the potential of a global virus as deadly as COVID-19.

Media and communication are obviously central to both the failures of this imaginary as well as how this became activated over the first wave of COVID-19. The pieces in this subsection hint at some of the wider disruptions within Australian communication practices within the micro contexts of regional spaces, localised economies and other parts of Australian society as core facets of everyday life became suspended.

Marianne Clark, in ‘Signs, Bodies, and Beaches in Pandemic Times’, offers an autoethnographic analysis of signage surrounding Sydney beaches, one of any number of everyday disruptions which helped to bring home to Australians the significance of the virus. As Clarke notes, within Australian culture, beaches represent a particular kind of egalitarian public commons, prime locations for leisure and personal freedom. Her commentary centres on the disconcerting and unexpected regulation and governance of these spaces.

Katherine Kirkwood and Terry Flew, in ‘The Impact of COVID-19 on Cultural Tourism: Art, Culture and Communication in Four Regional Queensland Sites’, discuss the shock of absent tourist populations in the centres of Cairns, the Gold Coast, Central West and the Sunshine Coast. While Queensland was not a key centre for proliferating cases of the virus, a suspension of travel between the Australian states quickly revealed the fragility of local economies based on strong seasonal domestic tourism. In the case of Queensland regional centres, local tourism serves as a supplement to a broader dependence upon a flow of international visitors attracted to the state’s unique landscape and cultural experiences.

A key frustration for many local enterprises, in many cases with serious implications for their continued survival, was their absence from the list of more ‘essential’ industries and sectors of economic activity which could enjoy more immediate federal government support during the unfolding crisis. The norm for most economic sectors was an ongoing shockwave as the impact of a loss of consumers became prolonged, and for some their underlying vulnerabilities meant they became open to more transformative changes.

Hess and Waller, in ‘Local Journalism and Coronavirus: Connections, Comparisons and Cure’, look at the fallout for regional newspapers from COVID-19, within a broader context of long-standing painful adjustments to digital news environment that is siphoning resources away from small-scale, regional news practices. These authors look at the media coverage of these developments, using an analogic approach.

In ‘Changing Tides: The Impact of Crisis on Advertising’, Cameron Jenyns offers a commentary on the challenges which the advertising industry faced in developing a more authentic style of address tailored to suit the current crisis, where existing appeals to individual consumerism now generated the wrong connotations.

This is followed by two pieces looking at aspects of mental health within media frames. Shelley Brunt and Kat Nelligan, ‘The Australian Music Industry’s Mental Health Crisis: Media Narratives During the Coronavirus Pandemic’, focus on messages being communicated to practitioners within another industry under severe stress, as live music venues were shuttered in the early months of the COVID crisis in Australia. They provide an initial typology of narratives surfacing across a range of media engaging with mental health issues of music industry personnel.

In ‘Communicating About Suicide During a Global Pandemic: Impact on Journalists and Media Audiences’ Elizabeth Paton et al. explore the stresses for working journalists during the crisis, both in terms of their personal exposure in developing news stories, and the challenges of operating in an environment in which there has been an existing crisis of trust in these kinds of institutions among some sections of Australian society.

The ABC was one such institution, already the focus of many years of political debate before the crisis, with its core functions and continued funding being challenged on a regular basis. Jessica Balanzategui et al., in ‘“What Would Bandit Do?”: The Educational Role of Australian Children’s Television During the COVID-19 Pandemic and Beyond’, focus on messages provided through television for children and their parents. Their commentary explores the distinctive pedagogical role which television has played in the past for local children and offers some initial observations of how this evolved in the first months of the COVID-19 crisis.

In ‘Bluey, Requestival, Play School And ME@Home- The ABC (Kids) of Communication Cultures During Lockdown’, Liz Giuffre explores in greater detail the specialist material produced for children viewers and listeners, designed to acknowledge and address some of the anxieties generated around uncertainties for how the virus was transmitted and the everyday health practices that needed to be adhered to for the safety of others.

The distinctive nature of the virus, which initially appeared to be particularly dangerous for elderly and those with existing health conditions, provided a basis for conflicting anxieties around the everyday needs and behaviours of children. Inevitably, this meant schools themselves became sites for social, economic and political debates over the appropriateness of a series of state and federal government anti-viral measures.

Two articles in this subsection focus on aspects of the complexities of public health communication involving stakeholders in school education. Schools themselves serve as an intersection point between key parts of the working population (as parents, carers and educators), uncertainties over whether classrooms and playgrounds were serving as sites for transmission of the virus and the particular challenges for educators themselves in adapting to more socially distanced (and then rapidly completely online) forms of teaching practice.

Lee-Ann Ewing and Huy Vu, in ‘Navigating ‘Home Schooling’ During COVID-19: Australian Public Response on Twitter’, provide a focused analysis of the response of Twitter users to the health directives and other measures across educational institutions generated within these circumstances. And in ‘The Role of Government’s “Owned Media” in Fostering Cultural Inclusion: A Case Study of the NSW Department of Education’s Online and Social Media During COVID-19’, Lauren Gorfinkel et al. engage specifically with the communication practice of the New South Wales Department of Education, providing an enquiry of this institution’s challenges in maintaining structured and meaningful connections with parents and carers in the multilingual context of Sydney.

In the last piece within this subsection, we offer an article which highlights that there were unexpected benefits enjoyed by at least some national figures during the COVID-19 first wave in Australia. Kurt Sengul’s commentary, ‘Never Let a Good Crisis go to Waste: Pauline Hanson’s Exploitation of COVID-19 on Facebook’, outlines the manner in which this well-known populist political figure used the initial uncertainties around COVID-19 to bolster her own anti-immigration anti-globalisation political platform.

## Media and pandemic

This second subsection ranges further afield, bringing in material which considers larger concerns of how different technologies, platforms and media practices are intersecting with reconfigured social spaces associated with life during a pandemic

Catherine et al. use the lens of ‘disaster capitalism’ to view the manner in which social media influencers (SMIs), one sector of the digital economy, responded strategically to the initial wave of COVID-19, in ‘Capitalising on Chaos – Exploring the Impact and Future of Social Media Influencer Engagement During and Beyond the COVID-19 Crisis’. Their analysis considers the potential of SMIs as agents for public health information.

Crystal Abidin et al. look at news discourse around the impact of COVID-19 on influencers, taking examples from four countries across the Asia-Pacific. In ‘Influencers and COVID-19: Reviewing Key Issues in Press Coverage Across Australia, China, Japan, and South Korea’, they focus on reportage drawing upon existing distinctions within localised identities around influencers and their economic and social aspects of their role significance, and how these are refracted through a COVID lens by localised news services.

A well-reported impact of COVID-19 was a rapid increase in demand for technologies to support socially distanced and dispersed workplaces, educational and health services and a host of online leisure and entertainment activities. For many this meant a deeper immersion into information proliferating through social media platforms, increased preference for video streaming platforms and most notably a dependence on the use of video conferencing services. Ash Watson et al. report on video ethnographic research into how users talk about such technology use and the implications on notions of intimacy and sociality, in ‘Enacting Intimacy and Sociality at a Distance in the COVID-19 Crisis: The Sociomaterialities of Home-Based Communication Technologies’.

All technologies have affordances which favour particular forms of engagement and communication over others and tend to be operated in ways which favour some social groups over others. During a health crisis, different levels of access to crucial information about what everyday practices should be adopted to minimise the risk of infection, and how to adapt individual behaviour in relation to others, obviously has serious implications for the health of different groups within society. Nhamo Mhiripiri and Ratidzo Midzi, in Fighting for Survival: Persons with Disabilities’ Activism for the Mediatization of Covid-19 Information’, provide insight into the role television served in efforts to communicate health information for people with disabilities in United States, United Kingdom, Zimbabwe and New Zealand.

In ‘Analysis of the Use of Memes as an Exponent of Collective Coping During COVID-19’ in Puerto Rico, Jose Flecha-Ortiz et al. examine memes which circulated in response to the uncertainties around personal behavioural changes demanded by COVID-19. Their analysis uses Collective Coping Theory to explore the possibilities of considering memes as a cultural mechanism for addressing users’ anxieties and processing different ways of coping with its impact.

Benjamin Mathews et al. provide an overview of the current state of XR as a field of emerging communication technologies which may become more integral to everyday life in an era of social distancing. In ‘Crisis and Extended Realities: Remote Presence in the Time of COVID-19’, they provide some reflections on the technical, social and ethical challenges of using immersive technologies and platforms to foster more socially engaging encounters than video conferencing has been able to provide.

Finally, within this concurrent Extraordinary issue, we have two pieces which engage with how issues of datafication have intersected with state responses to the emerging health emergency. Dennis Nguyen’s commentary, ‘Mediatization and Datafication in the Global COVID-19 Pandemic’, addresses the necessity for data literacy as a crucial element of how individuals and social collectives engage with the implications of health and government responses to the virus. The broader context for this discussion includes global efforts to use various forms of self-tracking and surveillance mechanisms to aid in quick responses by health authorities to localised viral outbreaks. Once embedded there is the possibility that such mechanisms will become naturalised as a governance tool, and hence it will become difficult for citizens to argue for their extraction once emergency conditions end.

In this frame, Fan Yang, et al., in ‘COVID-19, Health Code, COVIDSafe, Covid Tracer, platforms, public health surveillance, privacy, citizenship’, provide an initial comparison between China’s Health Code on WeChat and Alipay, Australia’s COVIDSafe and New Zealand’s Covid Tracer. They emphasise how each tracking approach promotes an individualising of responsibility for collective public health with uncertain future implications.

The two parts of our Extraordinary Issue offer a first slice of research issues which are likely to inform research agendas and strategies which will extend beyond the current crisis and its immediate aftermath. COVID-19 has prompted the emergence of a rapid reorganisation of key facets of social, economic and political life, often hastily enabled to service a population suddenly asked to stay at home, work and study remotely and organise everyday life through technology platforms in ways which highlighted the value of everyday human connections. The tension between an increased efficacy in the means of engaging with the world purely through screen-based or other media, and the recognition of what is lost if everyone were compelled to interact in this way for extended periods, offers implications for media and communication which are likely to play out over the next few years. Research into all aspects of how mediatisation operates within different parts of societies globally, and what these mean for the digitally saturated as well as the disenfranchised, will be essential to properly surface and make sense of such fundamental characteristics of the COVID-19 era.

